# Hygiene and food safety practices among mothers as predictors of diarrhea risk in toddlers in Purwawinangun Village, West Java, Indonesia

**DOI:** 10.3389/fpubh.2025.1530828

**Published:** 2025-03-21

**Authors:** Suparmi Suparmi, Muhammad Faris Sasman, Ratnawati Ratnawati, Ninik Rustanti

**Affiliations:** ^1^Faculty of Medicine, Department of Biology, Universitas Islam Sultan Agung, Semarang, Indonesia; ^2^Faculty of Medicine, Universitas Islam Sultan Agung, Semarang, Indonesia; ^3^Faculty of Medicine, Department of Public Health, Universitas Islam Sultan Agung, Semarang, Indonesia; ^4^Faculty of Medicine, Department of Nutrition, Universitas Diponegoro, Semarang, Indonesia; ^5^Faculty of Medicine, Center of Nutrition Research (CENURE), Universitas Diponegoro, Semarang, Indonesia

**Keywords:** diarrhea, food safety, personal hygiene, toddlers, risk factor

## Abstract

**Introduction:**

While the factors contributing to diarrhea in children are well established, more literature on the influence of food hygiene practices on diarrhea in low-socioeconomic urban communities in Indonesia is still needed. This study investigated specific risk factors for toddlers’ diarrhea related to mothers’ personal hygiene and food safety practices.

**Methods:**

It utilized a cross-sectional design with consecutive sampling, involving 36 mothers with toddlers in Purwawinangun Village, Cirebon, West Java, Indonesia. Hygiene and food safety practices, as well as diarrhea incidence, were assessed using a questionnaire.

**Results and discussion:**

In a bivariate analysis using Fisher’s Exact Test, personal hygiene practices [prevalence rate (PR) = 3.50; 95% confidence interval (CI) = 11.454–33.696] were significantly associated (*p* < 0.05) with an increased risk of diarrhea. Regarding mothers’ food safety practices, children of those with poor food safety were significantly associated (*p* < 0.05) with an increased risk of diarrhea compared to children of mothers who adhered to food safety guidelines (PR = 4.20; 95% CI = 12.127–89.524).

**Conclusions:**

The risk of diarrhea in children can be mitigated by improving mothers’ hygiene behaviors and food safety practices. Mothers, especially those living in villages with limited water access, need ongoing education on the importance of food safety. To promote this practice, the local government can implement health initiatives to prevent diarrhea in children.

## Introduction

Food safety is a crucial factor influencing public health, especially in developing areas where food handling, preparation, and storage methods may be insufficient (1, 2). Unsafe food is a primary contributor to diarrhea, a severe yet prevalent health concern in young children that can lead to malnutrition, stunting, developmental delays, and, in severe instances, death. In rural and semi-rural regions, these hazards are sometimes exacerbated by restricted access to healthcare, insufficient sanitation, and a lack of understanding of hygienic habits (3). Vulnerable populations, such as toddlers with growing immune systems and specific dietary requirements, face heightened health risks from exposure to contaminated food, which can result in recurrent episodes of diarrhea (4).

Diarrhea is characterized by the symptom of an infection in the intestinal tract, which may be attributed to various bacterial, viral, and parasitic agents. The disease is transmitted via contaminated food, drinking water, or person-to-person contact due to inadequate hygiene. Diarrheal illness ranks as the third primary cause of mortality in children under five years of age, resulting in around 443,832 fatalities annually. The latest data from the 2020 Indonesian Nutrition Status Survey shows that the prevalence of diarrhea is 9.8%. According to the 2020 Indonesia Health Profile data, diarrhea is a significant contributor to deaths among children aged 29 days to 11 months, causing 14.5% of deaths. Among children under five (12–59 months), the mortality rate due to diarrhea is 4.55% (5). In 2022, diarrhea cases across all ages in South Java that were treated amounted to 33,049 people or 53.3% of the estimated 61,995 cases. Diarrhea cases in children under five who received treatment totaled 13,625 cases, which is 43.6% of the estimated 31,253 toddler diarrhea cases (6).

Although the causes of diarrhea in children are well documented, there remains a need for more research on how food hygiene practices affect diarrhea in low socioeconomic urban communities in Indonesia (7). In East Jakarta, poor food hygiene practices among mothers have been linked to diarrhea in children under two years old. Enhancing mothers’ knowledge and practices related to food and personal hygiene could help prevent diarrhea in children aged 6 to 36 months (8). Factors such as water, sanitation, personal hygiene practices, knowledge, and awareness contribute to the prevalence of undernutrition and diarrhea in urban slums like those in Bandung, Indonesia (9). This study examines specific risk factors related to food safety and personal hygiene in Purwawinangun Village, exploring their association with the high rates of diarrhea among toddlers. By focusing on this vulnerable age group, the research intends to identify modifiable risk factors and recommend effective interventions to mitigate health risks. Purwawinangun Village, located in Cirebon, West Java, serves as a rural area in Indonesia where food safety issues are particularly concerning.

## Materials and methods

### Study design, population, and sample

This research is an analytical observational study with a cross-sectional design. Thirty-six pairs of mothers and their toddlers at Purwawinangun, Suranenggala, Cirebon, West Java, Indonesia, were involved in this study—the inclusion criteria required having at least one toddler aged 1 to 4 years. The exclusion criteria included toddlers with congenital disabilities, those diagnosed with chronic diseases, and missing written consent from the mothers. The sample size was 36, calculated using the following equation: where n is the minimum sample size required in the study, N is population size (70), Z is the area under the curve corresponding to the desired confidence interval used in this study, that is, 95% CI (1.96), p is the prediction of diarrhea in Indonesia (95%), and d is the precision (difference between the sample mean and population mean ± 5%).


$$n=\frac{N.Z^{2}1-\frac{\alpha }{2}.p.q}{d^{2}\left(N-1\right)+Z^{2}1-\frac{\alpha }{2}.p.q}$$

### Data collection tool and measurement

Data collection was performed from September 1 to 30, 2021. All research instruments, including the consent forms and questionnaires, were available in Indonesian. The mothers completed the questionnaire, and we provided direct guidance when necessary.

The self-administered structured questionnaire was developed to refer to peer-reviewed literature on hygiene and food safety measures (10, 11) with some modifications. The questionnaire consisted of sociodemographic characteristics (age, occupation, education, toddler age, and sex), personal hygiene practices, attitudes toward food safety, and incidence of diarrhea. A practices-level questionnaire on personal hygiene included 20 questions regarding attitudes during food preparation for their families (Table 1), with responses of Never (Score = 1), Seldom (Score = 2), Often (Score = 3), or Always (Score = 4). The level of attitude toward personal hygiene is categorized as Better if the total score is 41–80 and Poor if the total score is 1–40. Food safety attitudes were measured through a questionnaire with 10 questions using the same response scale. The level of attitude toward food safety is categorized as Better if the total score is 21–40 and Poor if the total score is 1–20 (Table 2). The occurrence of diarrhea was analyzed using direct questions (Yes or No responses) about whether their child had diarrhea within the last 6 months. Before conducting the research, the self-designed questionnaire was administered to 36 randomly selected mothers for comprehension testing to ensure validity and reliability. The Pearson Product Moment validity test showed that the Pearson correlation was >0.329 (r value for 36 respondents), while the reliability test demonstrated that Cronbach’s Alpha values for all items were > 0.329. Therefore, no significant changes were made due to the preliminary examination. The occurrence of toddler diarrhea was assessed using a three-question questionnaire (Table 3). Each question can be answered with “Yes” or “No.”

**Table 1:** The questions on practice regarding personal hygiene to mothers (*n* = 36).

No	Question
1.	I handle/serve food in a healthy condition or when I am not sick.
2.	I touch food using clean clothing.
3.	I do not smoke during food processing.
4.	I do not have open wounds while handling food.
5.	I keep my nails short and clean.
6.	I do not wear jewelry while handling food.
7.	I tie my hair or wear a head covering while handling food.
8.	I do not eat or drink while preparing food.
9.	I do not scratch my body while preparing food.
10.	I do not engage in conversation while preparing food.
11.	I do not sneeze or cough toward the food.
12.	I do not spit while in the food preparation area.
13.	I always wash my hands before preparing food.
14.	I do not scratch my body while preparing food.
15.	I dry my hands with a towel before serving food.
16.	I use a spoon when tasting cooked food.
17.	I do not touch food directly with my hands when serving; I use a spoon or other utensil.
18.	Perishable ingredients like milk or coconut milk are stored in the refrigerator or an icebox.
19.	I use the same cooking oil no more than three times.
20.	I always place food/drinks in a clean container (not lined with newspapers or other materials that might cause contamination).

**Table 2:** The questions on attitudes regarding food safety to mothers (*n* = 36).

No	Question
1.	While serving food, I don a head covering, an apron, and gloves.
2.	Soiled utensils are initially immersed in detergent, subsequently rinsed with clean water, and ultimately rinsed with hot water.
3.	I use a distinct knife to slice raw components and another to prepare dishes.
4.	Clean dishes and utensils are promptly dried with a cloth.
5.	The dishwashing sink remains uncleaned until it becomes filthy.
6.	It is essential to wash hands before and after preparing or serving food and beverages.
7.	I refrain from sneezing or speaking when preparing and serving food.
8.	I refrain from using recycled plastic to wrap food.
9.	Ingredients do not require cleaning before slicing or cutting, provided they are adequately cooked.
10.	I consistently scrutinize the nutritional labels and expiration dates on food items.

**Table 3:** The questions on symptoms of diarrhea (*n* = 36).

No	Respondent characteristic
1.	Has your child recently suffered diarrhea?
2.	Does your toddler have bowel movements exceeding three times in a single day?
3.	Is your toddler’s feces liquid (soft), with or without mucus and blood?

### Data analysis

Data were analyzed using IBM SPSS Statistics version 25.0 for Windows. The primary outcome is counting data, which represents diarrhea occurrence among toddlers. A bivariate analysis of the Fisher’s Exact Test was conducted to explore the relationship between practices toward personal hygiene and food safety and the risk of diarrhea. Due to some subgroups’ relatively small sample size, Fisher’s exact test was deemed more appropriate to ensure accurate and reliable results, as it is better suited for analyzing categorical data with low expected frequencies.

## Results

This study involved 36 mothers, of whom 44.4% were older than 20, 58.3% graduated from Senior High School, and 86.1% were homemakers. Of all mothers with 36 children, 55.6% had female children aged 1 to 4 (Table 4).

**Table 4:** Sociodemographic characteristics of respondents (*n* = 36).

Respondent characteristic	*n* (%)
Age	
> 20 years old	16 (44.4)
> 30 years old	17 (47.2)
> 40 years old	3 (8.3)
Education level	
Did not finish elementary school	3 (8.3)
Completed elementary school	2 (5.6)
Completed middle school	7 (19.4)
Completed high school	21 (58.3)
Bachelor’s degree	3 (8.3)
Occupation	
Housewife	31 (86.1)
Vendor	3 (8.3)
Business/Self-employed	2 (5.6)
Toddler’s gender	
Male	16 (44.4)
Female	20 (55.6)
Toddler’s age	
1 year old	8 (22.2)
2 years old	11 (30.6)
3 years old	11 (30.6)
4 years old	6 (16.7)
Personal hygiene practices	
Better	24 (66.7)
Poor	12 (33.3)
Food safety practices	
Better	28 (77.8)
Poor	8(22.2)
Occurrence of diarrhea	
Yes	11 (30.6)
No	25 (69.4)

Diarrhea occurred in 69.4% of toddlers. The prevalence of diarrhea among toddlers varied significantly between those with poor and better hygiene behaviors of their mothers (*p* < 0.05). Six out of 36 toddlers whose mothers had poor food safety practices experienced diarrhea, while the prevalence was lower in toddlers whose mothers practiced better food safety (5 out of 36 toddlers) (Table 5). Mothers’ hygiene practices significantly correlated with diarrhea in toddlers, yielding a prevalence ratio (PR = 3.50, 95% CI: 11.454–33.696). Mothers’ attitudes toward food safety were significantly associated with diarrhea in toddlers (PR = 4.20, 95% CI: 2.127–89.524).

**Table 5:** The relationship between behavior toward personal hygiene and food safety practices on the occurrence of diarrhea.

Variables	Diarrhea [*n*, (%)]		p*	PR (CI)
	Yes	No		
Personal hygiene practices			<0.05	3.50 (11.454–33.696)
Poor	7 (58.3)	5 (41.7)		
Better	4 (16.7)	20 (83,3)		
Food safety practices			<0.05	4.20 (2.127–89.524)
Poor	6 (75.0)	2 (25.0)		
Better	5 (17.9)	23 (82.1)		

*Bivariate analysis of the Fisher exact test.

## Discussion

Comprehending the role of food safety procedures in child health within this community would illuminate broader public health implications and guide targeted health programs aimed at enhancing food hygiene standards and mitigating illness transmission in comparable rural environments. Our study indicates that the risk of having diarrhea is higher in children aged <5 years whose mothers had poor personal hygiene and food safety practices. Children who lived in houses with mothers who practiced better personal hygiene and food safety had a lower risk of diarrhea. This finding is consistent with existing literature suggesting that inadequate food safety and poor hygiene practices are key risk factors for diarrhea, particularly in areas with limited sanitation and health education access (12–14).

The association between the hygiene habits of mothers and toddler diarrhea was reinforced by a significant prevalence ratio (PR = 3.50, 95% CI: 11.454–33.696). Toddlers whose mothers exhibited poor hygiene practices were 3.5 times more susceptible to diarrhea than those whose mothers maintained superior hygiene standards. These findings correspond with international health research highlighting the importance of maternal hygiene in mitigating the spread of germs responsible for diarrheal illnesses (15). Effective handwashing techniques, utilization of clean water, teaching germ theory, and comprehensive health interventions can markedly decrease the prevalence of diarrhea in children (12, 15–17). Enhancing hygiene practices and reducing diarrhea risk are significantly influenced by education and the elevation of mothers’ economic level (18). Our finding on diarrhea prevalence is consistent with Agustina *et al.* (2014) that the poor food-hygiene practice score was significantly associated with more diarrhea among children aged <2 years, although overall, there was no relation to the prevalence of diarrhea among children under five urban area of Jatinegara, East Jakarta district, Indonesia (7). This finding can be attributed to the reality that weaning foods for young children, when prepared in unhygienic conditions, often become contaminated with pathogens, representing a significant risk factor for the transmission of diarrhea (19). Nonetheless, our investigation could not establish a link between the contamination of weaning foods and diarrhea, primarily due to the absence of an evaluation of specific enteropathogens. While foodborne infection is the primary mode of transmission for gastrointestinal infections in developed nations, the extent of its impact on diarrhea prevalence in low-income areas remains ambiguous (20).

In our study, we evaluated various food hygiene practices, including the hand-washing habits of mothers, food preparation methods (such as holding times and reheating practices), and the use of clean utensils for eating or cooking. While these factors were linked to potential sources of foodborne transmission, they did not demonstrate statistically significant associations with diarrhea. Hand washing is the most thoroughly examined hygiene practice with reliable evidence in developing countries (21). In households with low socioeconomic status, the lack of fundamental sanitation facilities can result in inadequate food hygiene and sanitation practices, thus causing the risk of food- or water-borne transmission (22, 23).

The study identified a substantial correlation between mothers’ views on food safety and the incidence of diarrhea in toddlers, with a prevalence ratio of 4.20 (95% CI: 2.127–89.524). This indicates that mothers’ perceptions and prioritization of food safety directly affect their children’s health. Mothers exhibiting a passive or indifferent stance on food safety may unintentionally expose their children to hazardous food handling habits, increasing the risk of diarrhea. These findings align with prior studies that associate maternal attitudes and knowledge with child health outcomes, particularly in preventing foodborne (24).

The significant incidence of diarrhea in toddlers emphasizes the necessity for focused interventions that enhance mother food safety awareness and practices. Educational initiatives emphasizing appropriate food handling, storage, and hygiene may significantly reduce the incidence of diarrhea in young infants. The results indicate that maternal attitudes must be targeted through behavioral change communication tactics, as enhancing mothers’ awareness and beliefs regarding food safety could directly benefit their children’s health. However, diarrhea is a multifactorial condition influenced by environmental, behavioral, and socio-economic factors—climate, clean water, sanitation access, hygiene education and behavior, and antibiotic use. Prevention efforts should focus on increasing access to clean water, hygiene education, and wise antibiotic use to reduce the incidence of diarrhea. (11, 25)

This study was subject to several limitations. First, this study was a cross-sectional design; thus, it is difficult to determine whether mothers’ low personal hygiene practices and food safety preceded the diarrhea risk in their children. Second, data collection by questionnaire may have led to recall bias and misreporting of diarrhea incidence of their children in the last 6 months. Furthermore, unfortunately, we do not physically observe the behavior of toddlers, which has not been measured and could potentially contribute to diarrhea in young children. This study represents a village of Purwawinangun, Suranenggala, Cirebon, West Java, Indonesia, and thus is not representative of all mothers and children; therefore, further research with a larger sample size and broader geographical coverage is necessary, which might yield different conclusions from this study.

## Conclusion

The results of this study show that the risk of diarrhea in children can be prevented by improving mothers’ hygiene behaviors and food safety practices. Mothers, especially those living in villages with limited water access, need ongoing education on the importance of food safety. To enhance this practice, the local government can provide health promotion initiatives to prevent diarrhea in children.

## Data Availability

The original contributions presented in the study are included in the article/supplementary material, further inquiries can be directed to the corresponding author/s.

## References

[1] Akhtar S Sarker MR Hossain A. Microbiological food safety: a dilemma of developing societies. Crit Rev Microbiol. (2014) 40:348–59. doi: 10.3109/1040841X.2012.742036, PMID: 23173983

[2] Unnevehr L. Food safety in developing countries: moving beyond exports. Glob Food Sec. (2015) 4:24–9. doi: 10.1016/j.gfs.2014.12.001

[3] Chan M. Food safety must accompany food and nutrition security. Lancet. (2014) 384:1910–1. doi: 10.1016/S0140-6736(14)62037-7, PMID: 25467592

[4] Uddin IM Endres K Parvin T Bhuyian MSI Zohura F Masud J. Food hygiene and fecal contamination on the household compound are associated with increased pediatric diarrhea in urban Bangladesh (CHoBI7 program). Am J Trop Med Hyg. (2023) 108:524–9. doi: 10.4269/ajtmh.22-0129, PMID: 36746654 PMC9978565

[5] Kemenkes RI. Rencana Aksi Program Tahun 2020–2024 Direktorat Jendral Pencegahan dan Pengendalian Penyakit Kementrian Kesehatan Republik Indonesia. Jakarta: Ministry of Health (2022).

[6] Dinkes-KabupatenCirebon. Profil Kesehatan Cirebon 2022. (2020). 1–224. Available online at: https://diskes.jabarprov.go.id/informasipublik/unduh/dDZmNElJOWc4RnhhNTZDOG01bG1hZz09 (Accessed June 26, 2024)

[7] Agustina R Sari TP Satroamidjojo S Bovee-Oudenhoven IMJ Feskens EJM Kok FJ. Association of food-hygiene practices and diarrhea prevalence among Indonesian young children from low socioeconomic urban areas. BMC Public Health. (2013) 13:977. doi: 10.1186/1471-2458-13-977, PMID: 24138899 PMC3813984

[8] Usfar AA Iswarawanti DN Davelyna D Dillon D. Food and personal hygiene perceptions and practices among caregivers whose children have diarrhea: a qualitative study of urban mothers in Tangerang, Indonesia. J Nutr Educ Behav. (2010) 42:33–40. doi: 10.1016/j.jneb.2009.03.003, PMID: 20129187

[9] Otsuka Y Agestika L Widyarani SN Yamauchi T. Risk factors for undernutrition and diarrhea prevalence in an urban slum in Indonesia: focus on water, sanitation, and hygiene. Am J Trop Med Hyg. (2019) 100:727–32. doi: 10.4269/ajtmh.18-0063, PMID: 30693865 PMC6402924

[10] Merza SA Putri NL Isfandiari AM. Higiene Sebagai Faktor Risiko Diare Pada Balita di Puskesmas Mulyorejo, Surabaya. J Berk Epidemiol. (2024) 12:290–7. doi: 10.20473/jbe.V12I32024.290-297

[11] Jannah AA Suryani D Sukesi TW. Determinants of food safety practices among mothers of toddlers in Gedongkiwo Village, Mantrijeron, Yogyakarta. J Penelit Pendidik IPA. (2023) 9:324–31. doi: 10.29303/jppipa.v9iSpecialIssue.6161

[12] Komarulzaman A Smits J de Jong E. Clean water, sanitation and diarrhoea in Indonesia: effects of household and community factors. Glob Public Health. (2017) 12:1141–55. doi: 10.1080/17441692.2015.1127985, PMID: 26758565

[13] Pradana VN Suparmi S Ratnawati R. Personal hygiene, water availability, and environmental sanitation with the incidence of stunting in toddlers aged 6–59 months in the working area of the Singorojo I public health center, Kendal regency: personal Higiene, Ketersediaan air, dan Sanitasi li. Amerta Nutr. (2023) 7:421–6. doi: 10.20473/amnt.v7i3.2023.421-426

[14] Morse T Tilley E Chidziwisano K Malolo R Musaya J. Health outcomes of an integrated behaviour-Centred water, sanitation, hygiene and food safety intervention–a randomised before and after trial. Int J Environ Res Public Health. (2020) 17:1–19. doi: 10.3390/ijerph17082648, PMID: 32294881 PMC7215646

[15] Feachem RGA Hogan RC Merson MH. Diarrhoeal disease control: reviews of potential interventions. Bull World Health Organ. (1983) 61:637–40. PMID: 6354505 PMC2536145

[16] Ahmed NU Zeitlin MF. Assessment of the effects of teaching germ theory on changes in hygiene behaviors, cleanliness, and diarrheal incidence in rural Bangladesh. Int Q Community Health Educ. (1993) 14:283–97. doi: 10.2190/A4XQ-QFL6-CPVV-XJEJ, PMID: 20841012

[17] Sheth AN Russo ET Menon M Wannemuehler K Weinger M Kudzala AC. Impact of the integration of water treatment and handwashing incentives with antenatal services on hygiene practices of pregnant women in Malawi. Am Soc Trop Med Hyg. (2010) 83:1315–21. doi: 10.4269/ajtmh.2010.10-0211, PMID: 21118942 PMC2990052

[18] Santika NKA Efendi F Rachmawati PD Has EMM Kusnanto K Astutik E. Determinants of diarrhea among children under two years old in Indonesia. Child Youth Serv Rev. (2020) 111:104838. doi: 10.1016/j.childyouth.2020.104838

[19] Motarjemi Y Käferstein FK Moy GG Quevedo F. Contaminated weaning food: a major risk factor for diarrhoea and associated malnutrition. Bull World Health Organ. (1993) 71:79–92. PMID: 8440042 PMC2393433

[20] Cissé G. Food-borne and water-borne diseases under climate change in low- and middle-income countries: further efforts needed for reducing environmental health exposure risks. Acta Trop. (2019) 194:181–8. doi: 10.1016/j.actatropica.2019.03.012, PMID: 30946811 PMC7172250

[21] Hashi A Kumie A Gasana J. Hand washing with soap and WASH educational intervention reduces under-five childhood diarrhoea incidence in Jigjiga District, eastern Ethiopia: a community-based cluster randomized controlled trial. Prev Med Reports. (2017) 6:361–8. doi: 10.1016/j.pmedr.2017.04.011, PMID: 28507890 PMC5425344

[22] Daniel D. Contextual determinants of general household hygiene conditions in rural Indonesia. Int J Environ Res Public Health. (2021) 18:1–15. doi: 10.3390/ijerph182111064, PMID: 34769584 PMC8582855

[23] Mc Michael C. Water, sanitation and hygiene (WASH) in schools in low-income countries: a review of evidence of impact. Int J Environ Res Public Health. (2019) 16:1–21. doi: 10.3390/ijerph16030359, PMID: 30696023 PMC6388361

[24] Bertrand WE Walmus BF. Maternal knowledge, attitudes and practice as predictors of Diarrhoeal disease in young children. Int J Epidemiol. (1983) 12:205–10. doi: 10.1093/ije/12.2.205, PMID: 6874217

[25] Luni D Manurung I Bakoil M Junias M Gero S. Risk factors of Diarhea incidence in children aged 6-24 months at Mangulewa and Koeloda community health centers, Ngada, Kupang, East Nusa Tenggara. Strength Hosp Compet Improv Patient Satisf Better Heal Outcomes. (2019). doi: 10.26911/the6thicph.01.09

